# Can Hematological Findings of COVID-19 in Pediatric Patients Guide Physicians Regarding Clinical Severity?

**DOI:** 10.4274/tjh.galenos.2021.2021.0157

**Published:** 2021-08-25

**Authors:** Kamile Ötiken Arıkan, Şahika Şahinkaya, Elif Böncüoğlu, Elif Kıymet, Ela Cem, Aybüke Akaslan Kara, Nuri Bayram, İlker Devrim

**Affiliations:** 1 University of Health Sciences Turkey, İzmir Dr. Behçet Uz Children Hospital, Clinic of Pediatric Infectious Disease, İzmir, Turkey

**Keywords:** COVID-19, Hematological parameters, Clinical severity, Red cell distribution width, Lymphopenia

## To the Editor,

The coronavirus disease-19 (COVID-19) pandemic originated in December 2019 in the city of Wuhan, the capital of Hubei Province, China. The virus then spread to numerous other countries in Asia and by January 2020 infected patients were identified in Europe [1]. Children of all ages are susceptible to infection by severe acute respiratory syndrome-coronavirus-2, the causative agent. Most children have relatively mild clinical symptoms without fever or pneumonia [2,3,4,5,6,7,8].

We conducted a retrospective study at the University of Health Sciences Turkey, İzmir Dr. Behçet Uz Children’s Hospital between March 30 and October 31, 2020.

A total of 3878 pediatric patients were tested and 353 (9.1%) of them were diagnosed with COVID-19. Of these 353 children, 184 (52.1%) were male (52.1%) (female/male: 0.91). The median age of the patients was 9 years (range: 4 days to 17 years). Thirty-five (9.9%) patients had underlying diseases, most commonly a neurological disease (n=9). Regarding severity, 9 (2.5%), 293 (83%), 38 (10.8%), and 13 (3.7%) cases were diagnosed as asymptomatic, mild, moderate, and severe/critical, respectively. Neutropenia (47.9%) was the most common abnormal parameter in complete blood counts, followed by lymphocytosis (22.4%), lymphopenia (20.7%), leukopenia (9.1%), neutrophilia (6.5%), and thrombocytopenia (3.4%) (Table 1).

Neutropenia was statistically significantly more common in neonates (84.6%). Lymphocytosis and neutrophilia were statistically significantly more common in infants (75.9%, p<0.001 and 23.3%, p<0.001, respectively). Lymphopenia and leukopenia were statistically significantly more common in patients >11 years old (38.4%, p<0.001 and 15.2%, p=0.025, respectively). Patients older than 11 years of age were more often thrombocytopenic, but this finding was not statistically significant (p=0.17).

The neutrophil-to-lymphocyte ratio (NLR) was higher in severe/critical cases compared to cases of asymptomatic, mild, and moderate severity [median NLR values in asymptomatic, mild, moderate, and severe cases were as follows: 0.84 (range: 0.2-3), 1.12 (0.04-28), 1.32 (0.11-4.6), and 3.39 (0.23-10), respectively; p=0.25].

The platelet-to-lymphocyte ratio statistically significantly increased as age increased.

Lymphocyte-to-white blood cell ratio statistically significantly decreased as age increased and it was lower in severe/critical cases compared to cases of asymptomatic, mild, and moderate severity. Red cell distribution width (RDW) statistically significantly increased in severe cases (median values in asymptomatic, mild, moderate, and severe cases were as follows: 12.3 [range: 12-13.1], 12.9 [11.2-13.2], 12.9 [11.6-19.5], and 14.9 [13-19.6], respectively; p=0.005). Median serum ferritin and D-dimer were statistically significantly increased in severe cases. Increased serum D-dimer was found to increase the risk of disease severity 2.9-fold (95% confidence interval: 0.13-0.85, p=0.022).

In our findings, the NLR ratio was higher in severe/critical cases compared to cases of asymptomatic, mild, and moderate severity. Qin et al. [6] reported an increase in NLR in patients with severe disease compared to those without [8]. In our findings, RDW levels were also significantly higher in severe cases. In adult studies, it was concluded that elevated RDW at the time of hospital admission and an increase in RDW during hospitalization were associated with increased mortality risk for patients with COVID-19, compatible with our results [6,9,10].

We recommend that clinicians closely monitor leukocyte count, lymphocyte count, platelet count, serum D-dimer, serum ferritin, and RDW as markers for potential progression to critical illness.

## Figures and Tables

**Table 1:**
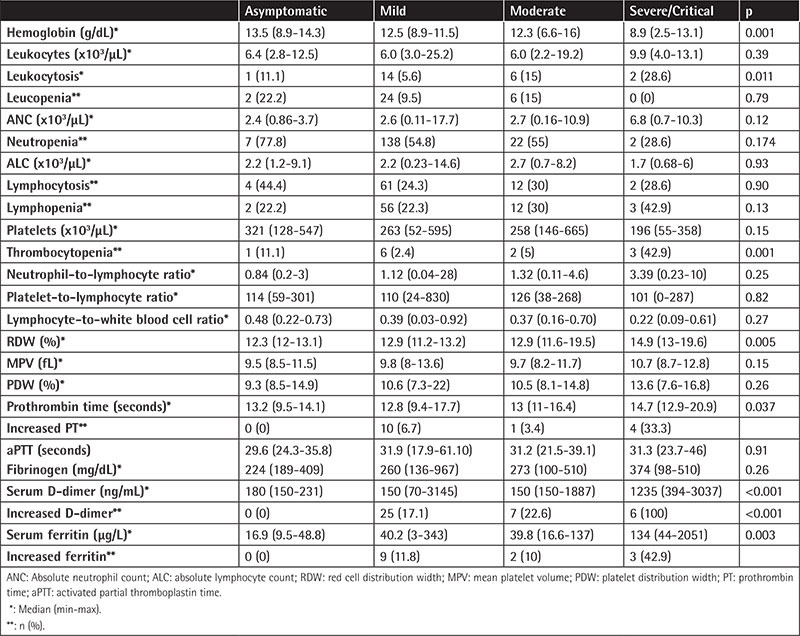
Comparisons of hematologic and biochemical parameters of patients according to disease severity. Asymptomatic Mild Moderate Severe/
